# Spatiotemporal Decision-AI for Species-Specific and Non-Lethal Management of Avian Predators in Aquaculture

**DOI:** 10.3390/s26206515

**Published:** 2026-10-14

**Authors:** Ronnie Serfa Juan, Julia Grace Lansdell, Joseph E. Gerken, Alison R. Gerken

**Affiliations:** 1 USDA-ARS Stored Product Insect and Engineering Research, Manhattan, KS 66502, USA; ronnie.serfajuan@usda.gov; 2 Department of Horticulture and Natural Resources, Kansas State University, Manhattan, KS 66502, USA; glansdell@ksu.edu (J.G.L.); gerkenje@ksu.edu (J.E.G.)

**Keywords:** aquaculture systems, avian monitoring, computer vision, decision-AI, non-lethal management, spatiotemporal modeling, species-specific risk assessment

## Abstract

**Highlights:**

- A computer vision model distinguished two predatory avian species, one non-predatory nuisance avian species, and a human reference class with high classification accuracy.
- Temporal and spatial variation from metadata provided preliminary risk assessment parameters.
- Classification coupled with targeted future deterrent deployment could better keep birds away from aquaculture ponds.
- An illustrative framework for using daily and seasonal patterns of bird activity can be used to better manage these predators.

**Abstract:**

Avian predation remains a persistent challenge in aquaculture, as conventional deterrents often lose effectiveness due to bird habituation and limited species specificity. Some methods may also unintentionally affect surrounding ecosystems. To address these limitations, this study presents a spatiotemporal Decision-AI approach for species-specific and non-lethal management of avian predators and nuisance birds by integrating visual intelligence with ecological context. The proposed framework integrates a YOLOv8-based detection model with a metadata extraction and spatiotemporal analysis pipeline that records species identity, population count, time, and location. YOLOv8 serves as the visual detection component, while the methodological contribution of the framework lies in transforming these detections into contextual ecological indicators through temporal occurrence analysis, species-specific weighting, and risk-based Decision-AI. This allows the system to transform raw image detections into meaningful ecological indicators that can guide management decisions. The study was evaluated using a dataset of more than 4500 high-resolution images collected from aquaculture sites across multiple U.S. locations. The proposed model achieved an overall classification accuracy of 97.2%, with precision and recall values of 95.8% and 96.4%, respectively. More importantly, the integration of detection results with temporal and spatial metadata enabled the identification of activity patterns, including peak visitation periods, species dominance trends, and location-specific risk levels. These insights can support targeted and non-lethal intervention strategies, such as optimizing deterrent timing and applying species-aware responses, while minimizing disturbance to non-target wildlife. By linking computer vision with ecological decision support, the proposed study moves beyond passive monitoring toward data-informed, adaptive management. This approach provides a scalable and interpretable pathway for improving aquaculture sustainability while maintaining ecological balance.

## 1. Introduction

Aquaculture plays an important role in global food production and economic sustainability; however, interactions between aquaculture facilities and avian wildlife remain an important operational challenge [1]. Fish-eating and opportunistic bird species can contribute to production losses through direct predation, disturbance of cultured fish, and potential contamination of production environments [2,3]. Species such as the great blue heron (*Ardea herodias*), great egret (*Ardea alba*), and other water-associated birds are frequently encountered around aquaculture facilities, making effective monitoring and management an important component of farm operations [4,5,6]. Conventional bird-management approaches include visual deterrents, acoustic devices, physical exclusion, and, in some circumstances, lethal control. Although these approaches can temporarily reduce bird activity, their effectiveness may decrease as birds habituate to repeatedly presented stimuli [7]. Physical exclusion methods can also be difficult to deploy across large or open-water production systems, while lethal approaches introduce additional ecological and ethical concerns [8,9]. Collectively, these limitations have increased interest in adaptive, species-specific, and non-lethal management approaches that can respond to observed bird activity rather than continuously applying the same deterrent strategy.

Advances in computer vision and deep learning provide an opportunity to support this transition. Convolutional neural networks and object-detection frameworks have increasingly been applied to wildlife monitoring because they can automate species recognition from large image collections while reducing reliance on manual image inspection [10,11,12]. Among object-detection architectures, the You Only Look Once (YOLO) family has progressed from early single-stage detection models toward increasingly efficient architectures capable of combining localization and classification within a unified framework. Successive YOLO generations have improved detection efficiency, feature representation, multi-scale recognition, and suitability for real-time or near-real-time applications. These characteristics make YOLO-based approaches particularly relevant to field monitoring, where wildlife may occupy relatively small portions of visually complex scenes [13].

However, accurate species detection alone does not fully address the information needs of ecological management. Many computer-vision systems primarily generate image-level outputs such as species labels, bounding boxes, and confidence scores, with comparatively limited integration of the temporal and spatial context in which detections occur. In aquaculture environments, knowing that a bird was detected is useful, but determining which species was present, how many individuals occurred, where the observation occurred, when the interaction took place, and whether the activity was isolated or recurrent provides substantially greater ecological and operational value. Integrating contextual metadata with visual detections therefore offers a pathway from image-level recognition toward spatiotemporal ecological interpretation. Metadata such as timestamps, camera identifiers, location, species identity, population count, and detection frequency can be used to characterize when and where avian interactions occur and whether particular species exhibit repeated or concentrated activity patterns. Although substantial progress has been made in automated wildlife recognition, a unified framework capable of supporting management decisions in aquaculture environments is lacking. To address this gap, this study proposes a spatiotemporal Decision-AI framework that integrates a YOLOv8-based object-detection module with structured temporal and spatial metadata. Rather than modifying the underlying YOLOv8 architecture, the proposed framework extends its detection outputs into a decision-oriented ecological analysis by incorporating species identity, population abundance, occurrence frequency, and species-specific ecological relevance.

## 2. Materials and Methods

**Figure 1:**
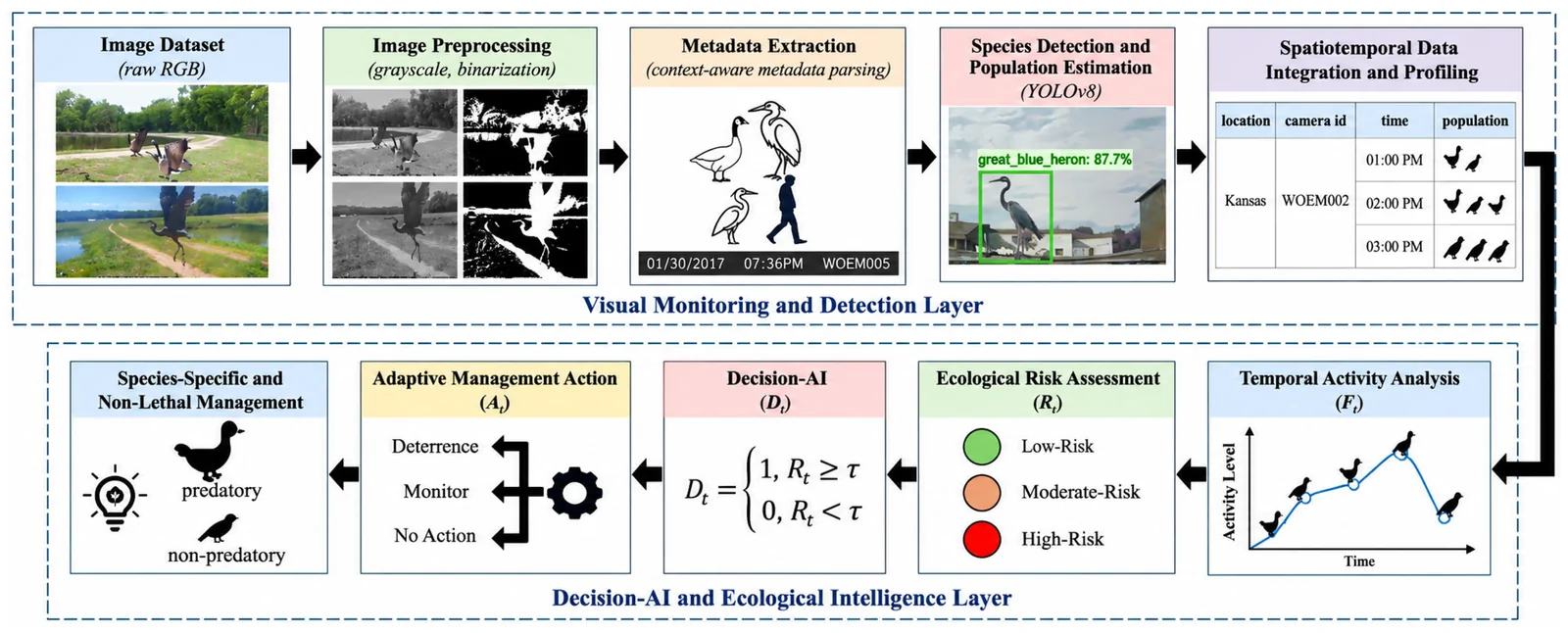
Proposed spatiotemporal Decision-AI method for species-specific and non-lethal management of avian predators in aquaculture environments. The architecture consists of a Visual Monitoring and Detection Layer and a Decision-AI and Ecological Intelligence Layer. Raw image observations are transformed through metadata extraction, species detection, spatiotemporal profiling, temporal activity analysis, ecological risk assessment, and adaptive decision-making to generate actionable management recommendations.

The complete workflow of the proposed system is illustrated in Figure 1. Unlike conventional avian monitoring systems that primarily focus on species detection, the proposed architecture integrates visual monitoring, ecological intelligence, and adaptive decision-making within a unified spatiotemporal Decision-AI system. The system is organized into two interconnected layers: (i) a Visual Monitoring and Detection Layer responsible for image acquisition, species recognition, and ecological data generation, and (ii) a Decision-AI and Ecological Intelligence Layer responsible for temporal activity analysis, ecological risk assessment, and adaptive management recommendations. Together, these layers transform raw image observations into actionable ecological intelligence that supports species-specific and non-lethal management of avian predators in aquaculture environments.

As shown in Figure 1, the workflow begins with image dataset acquisition, where raw RGB images captured from aquaculture surveillance systems serve as the primary input. These images undergo preprocessing operations including grayscale conversion and binarization to enhance image quality and facilitate downstream metadata extraction. The metadata extraction module subsequently identifies and parses contextual information embedded within each image, including timestamps and camera identifiers, enabling the generation of temporally and spatially referenced observations.

The processed images are then analyzed using the YOLOv8-based detection module, which performs species detection and population estimation. Detected observations are integrated with metadata records to create a structured ecological database containing species identity, abundance, location, camera information, and temporal attributes. This spatiotemporal data integration and profiling stage establishes the foundation for ecological interpretation by linking biological observations with their corresponding spatial and temporal context. To benchmark the performance of the proposed YOLOv8-based detection model framework, four widely adopted deep learning architectures, namely ResNet50, VGG16, MobileNetV2, and EfficientNet-B0, were implemented using the MATLAB (version RB 2024) Deep Learning Toolbox (MathWorks, Natick, MA, USA). Transfer learning was performed by replacing the final classification layer of each pretrained network with a four-class output corresponding to Great Blue Heron, Great Egret, Canada Goose, and Human. All models were trained using the same image dataset, identical preprocessing procedures, augmentation settings, optimizer configuration, and 70:30 training–testing partition to ensure a fair and unbiased performance comparison. Model performance was subsequently evaluated using overall accuracy, precision, and recall under the same testing conditions employed for the proposed YOLOv8-based detection model framework.

The second layer of the method focuses on ecological intelligence and decision support. Temporal activity analysis is first performed to characterize species occurrence patterns and behavioral trends over time. These temporal indicators are subsequently incorporated into an ecological risk assessment module, where species abundance, occurrence frequency, and species-specific ecological weighting factors are combined to estimate a risk score associated with observed avian activity.

The resulting risk information is forwarded to the Decision-AI module, which evaluates whether management intervention is warranted based on predefined ecological thresholds. Finally, the adaptive management action module generates species-specific recommendations ranging from monitoring-only responses to targeted non-lethal deterrence strategies. By distinguishing between predatory and non-predatory species, the proposed approach supports selective intervention while minimizing unnecessary disturbance to non-target wildlife, thereby promoting both aquaculture productivity and ecological sustainability.

### 2.1. Dataset, Tools, and Development Environment

This study utilized a curated dataset of 4500 high-resolution RGB images collected from aquaculture facilities in Kansas, Michigan, and Missouri. The dataset was balanced across sites, with approximately 1500 images per location, ensuring representative sampling of environmental conditions and species distribution. To ensure that the proposed method was evaluated under realistic aquaculture conditions, image data were collected from multiple commercial and research aquaculture facilities located in Kansas, Missouri, and Michigan from April to October 2024. These sites differ in pond geometry, facility layout, and surveillance coverage, providing diverse environmental conditions for evaluating species detection, metadata extraction, and spatiotemporal analysis. The cameras in Missouri covered seven ponds of various sizes ranging from approximately 8650 ft^2^ to 79,810 ft^2^. In Kansas there were six ponds, with five approximately 34,800 ft^2^ and one 28,565 ft^2^. In Michigan there were two ponds, approximately 34,057 ft^2^ and 47,437 ft^2^. Cameras were spaced evenly around the ponds to reduce overlap in image capture. Images were captured using stationary Browning cameras in JPEG format with a resolution of 3840 × 2160 pixels. Camera metadata, including timestamp and camera identification information, were embedded within the images and used to provide temporal and spatial context for subsequent analysis.

### 2.2. Image Processing and Metadata

All preprocessing and analysis procedures were implemented in MATLAB R2024b (MathWorks, Natick, MA, USA), utilizing the Image Processing Toolbox and Deep Learning Toolbox. The initial step involves isolating the region of interest (ROI) containing embedded metadata overlays. Each image included in the detection dataset was reviewed and assigned to one of the four target classes: great blue heron, great egret, Canada goose, or human. For object-detection training, individual target instances were annotated using bounding boxes enclosing the visible extent of each target. Each bounding box was assigned its corresponding class label and subsequently converted to the annotation format required by the YOLOv8 training pipeline. Images and their associated annotations were checked prior to model training to identify incorrect labels, incomplete bounding boxes, or inconsistent class assignments. Images containing multiple individuals were annotated separately so that each visible target instance could contribute to both species detection and population estimation. To enhance readability and extraction accuracy, the ROI undergoes grayscale conversion, adaptive thresholding, and morphological filtering.

The RGB images were converted to grayscale using the luminance weighting coefficients specified by the ITU-R BT.601 standard [14], expressed as:

(1) $$I_{gray}\;=\;0.299R\;+\;0.587G\;+\;0.114B$$

Adaptive thresholding and morphological operations are subsequently applied to enhance text visibility and isolate metadata regions.

A custom Image-Based Text Parsing (IBTP) module is then used to extract structured metadata, including timestamp, camera ID, and derived location labels. The metadata vector is defined as:

(2) $$M_{t}\;=\;f_{IBTP}\left(I_{morph}\right)\;=\;\left\{Timestamp_{t},CameraID_{t},Location_{t}\right\}\;\;$$

**Figure 2:**
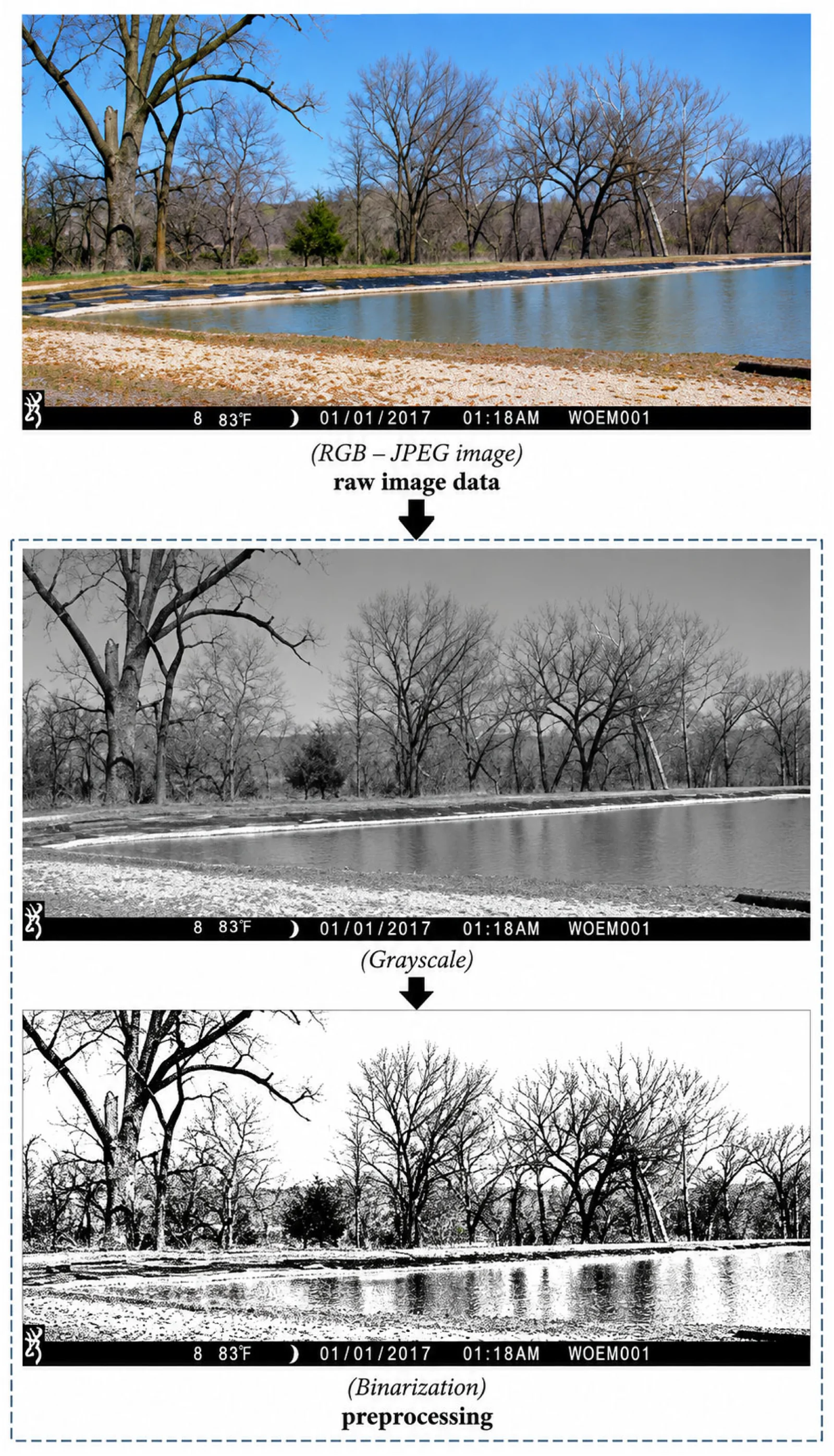
Metadata extraction workflow illustrating RGB image acquisition, grayscale transformation, and binarization preprocessing prior to automated timestamp and camera-ID parsing.

This process ensures temporal and spatial alignment of detections, forming the basis for downstream spatiotemporal analysis. As illustrated in Figure 2, the original RGB image is first converted to grayscale to reduce computational complexity while preserving relevant textual information. The grayscale image is subsequently binarized to enhance contrast between foreground text and background regions, facilitating more reliable extraction of timestamps and camera identifiers. This preprocessing stage establishes the foundation for generating temporally and spatially referenced observations used throughout the proposed Decision-AI system.

**Figure 3:**
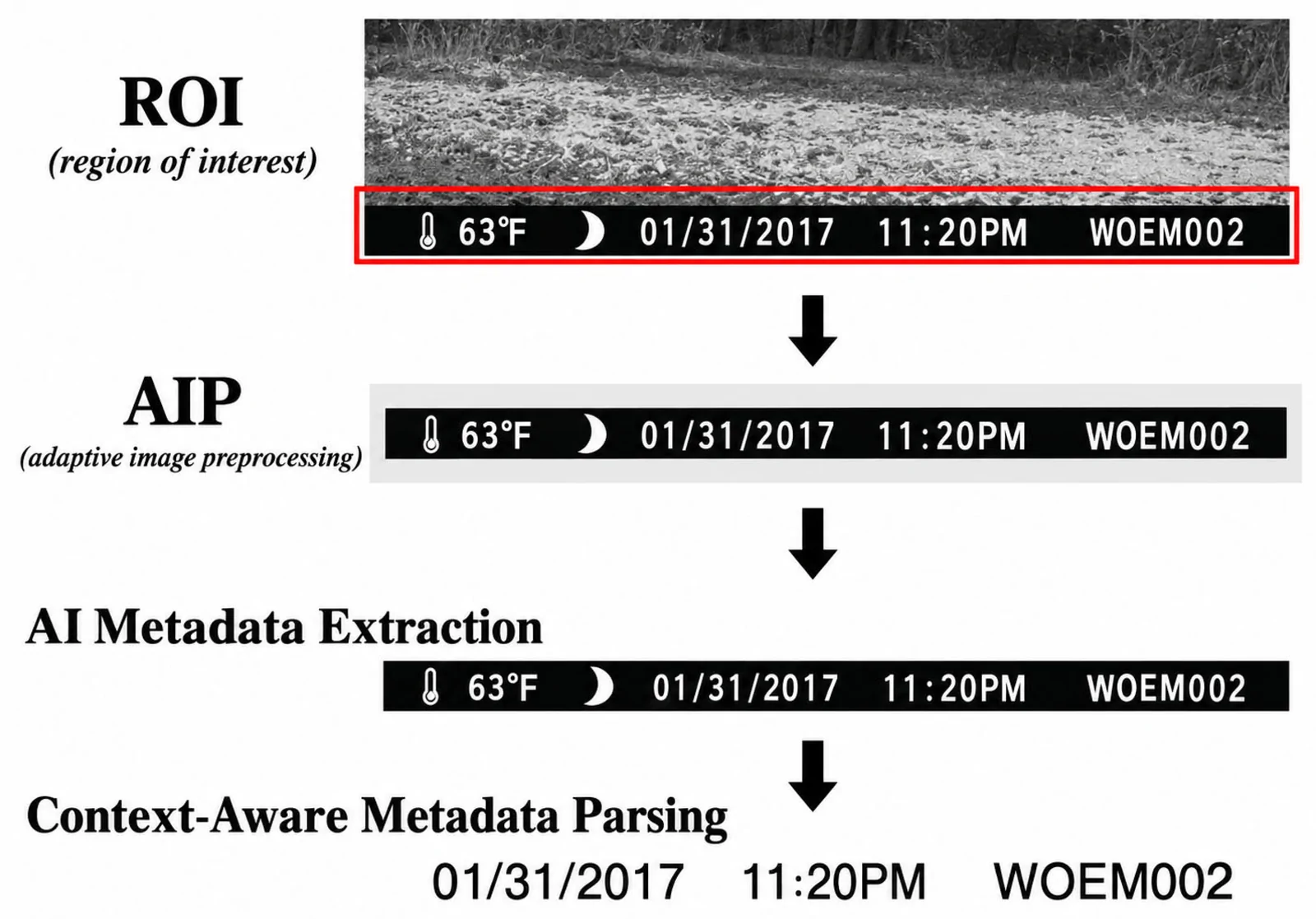
Context-aware metadata extraction workflow illustrating region-of-interest (ROI) localization (red box in top panel), adaptive image preprocessing (AIP), optical character recognition, and structured metadata generation. The extracted timestamp and camera identification information provide the spatial and temporal context required for spatiotemporal profiling and ecological intelligence analysis.

Following image preprocessing, the proposed method performs automated metadata extraction to recover contextual information embedded within surveillance images. This information includes environmental readings, timestamps, and camera identifiers that provide the spatial and temporal references necessary for ecological analysis. As shown in Figure 3, a region-of-interest (ROI) corresponding to the metadata overlay is first localized within the image. An adaptive image preprocessing (AIP) procedure is then applied to enhance text visibility and improve optical character recognition performance. The metadata extraction workflow was initially developed and validated entirely in MATLAB using the Image Processing Toolbox and the built-in Optical Character Recognition (OCR) function. During large-scale processing of millions of images, we also used a Python-based OCR (version pytesseract 0.3.13) implementation because it was significantly faster for bulk metadata extraction. Both implementations followed the same preprocessing and metadata parsing workflow. The extracted metadata are subsequently converted into a structured format through context-aware metadata parsing, producing standardized records that can be integrated with species detection results. This process enables each biological observation to be linked to a specific location and time, thereby supporting downstream spatiotemporal profiling, temporal activity analysis, and ecological risk assessment.

### 2.3. Species Detection and Population Estimation

**Figure 4:**
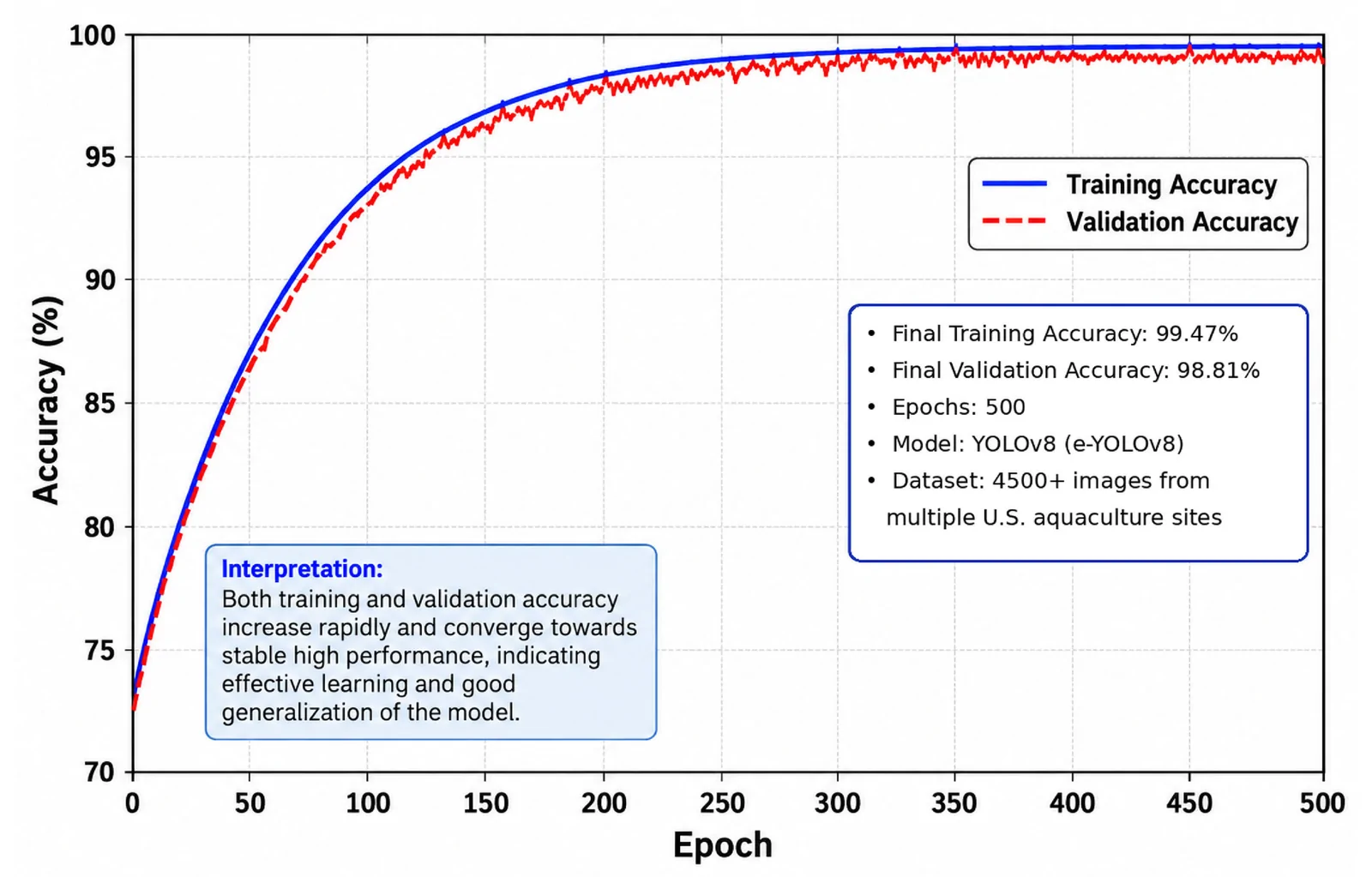
Training and validation accuracy curves of the YOLOv8-based detection model used within the proposed spatiotemporal Decision-AI framework.

**Figure 5:**
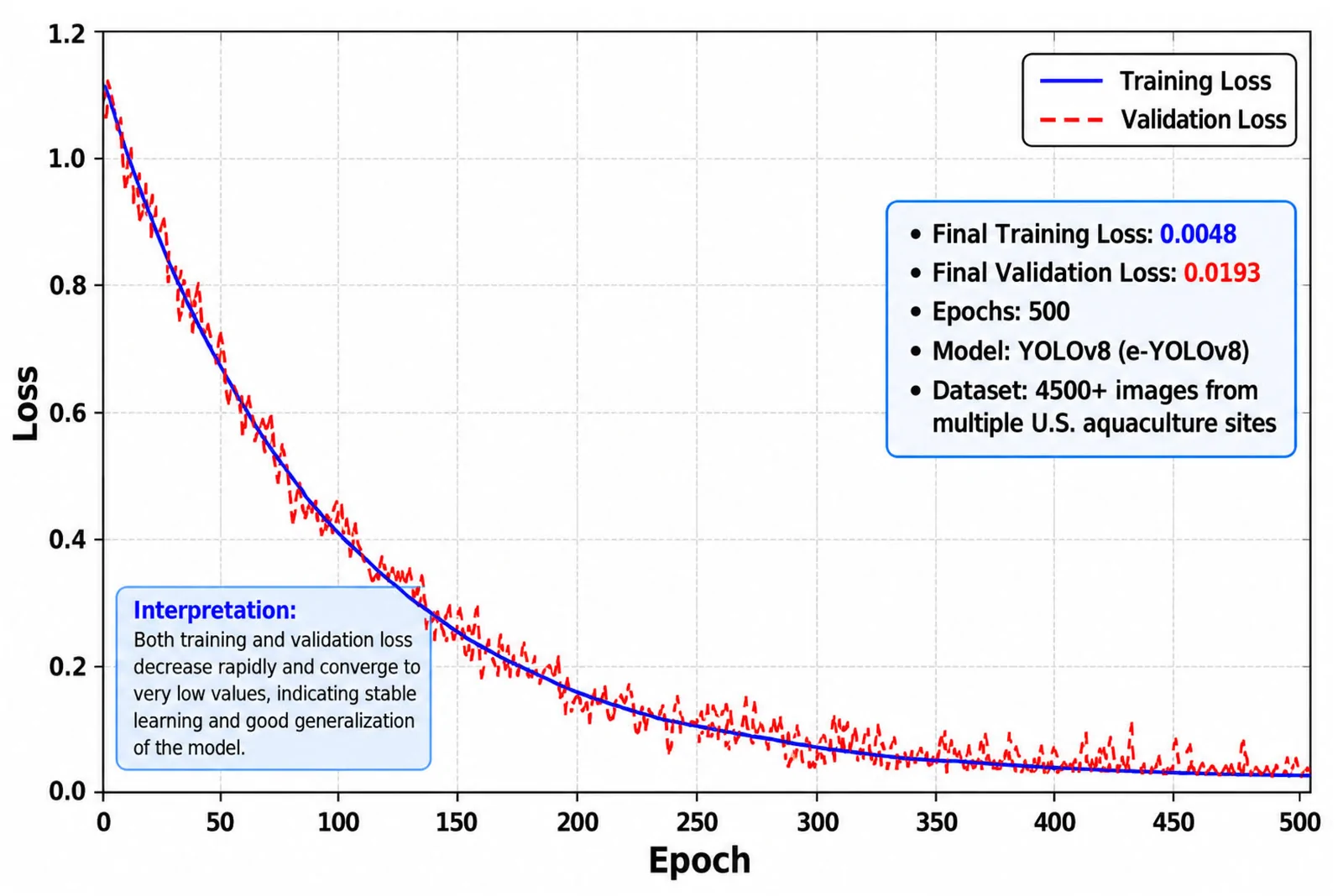
Training and validation loss curves of the YOLOv8-based detection model, illustrating stable optimization and convergence during model training.

Species detection was performed using a YOLOv8-based object-detection model. The underlying YOLOv8 network architecture was retained without structural modification; no new backbone, detection head, or loss function was introduced in this study. Instead, the model was trained for the specific aquaculture-monitoring application using four target classes: great blue heron, great egret, Canada goose, and human. The dataset was partitioned using a 70:30 training–testing split of the 4500 annotated images. The YOLOv8-based detector was trained for 100 epochs using an input image size of 640 × 640 pixels, a batch size of 16, and an initial learning rate of 0.001. Model optimization was performed using stochastic gradient descent (SGD) with Nesterov momentum, with a cosine learning-rate scheduler. During training, data augmentation was applied only to the training subset and included horizontal flipping and scale variation to increase variability in object orientation, position, and apparent size. The validation subset was not augmented. Training and validation metrics were recorded over 500 epochs. The curves shown in Figure 4 and Figure 5 represent the recorded epoch-level training history, and no post hoc smoothing, interpolation, or curve fitting was applied. The principal methodological extension of the proposed framework occurs after visual detection. Rather than modifying the internal YOLOv8 architecture, the detector outputs are integrated with temporal and spatial metadata, population estimates, species occurrence frequency, and species-specific ecological weighting. These combined outputs provide the input to the subsequent spatiotemporal risk-assessment and Decision-AI components described in Section 2.4, Section 2.5, Section 2.6 and Section 2.7.

For each image, the detector produced bounding boxes, predicted class labels, and associated confidence scores. Non-maximum suppression was applied using an intersection-over-union threshold of 0.5 to reduce overlapping detections, and population counts were subsequently derived by aggregating accepted detections for each class. Images associated with the same camera location and/or capture session were retained within a single partition to reduce the possibility that closely related or temporally adjacent observations were distributed across both training and testing subsets. This strategy was used to minimize potential data leakage and to provide a more realistic estimate of model performance on independent field observations.

In addition, aquaculture surveillance images are subject to environmental variability, including changes in illumination, reflective water surfaces, vegetation, background complexity, target scale, and partial occlusion. Because the dataset was collected from multiple facilities in Kansas, Missouri, and Michigan, these conditions were naturally represented to varying degrees across the image collection. Representative detections were qualitatively examined under different field conditions, including relatively uniform illumination, reflective water backgrounds, vegetation-rich scenes, distant targets, and partial occlusion. Because illumination, weather, and occlusion were not systematically recorded as independent variables for all images, this analysis should be interpreted as a qualitative field-condition assessment rather than a controlled robustness experiment. Future work should include condition-specific labels and stratified performance analysis to quantify model stability under different environmental and camera conditions. The YOLOv8-based detector remained capable of identifying the target classes across these representative conditions, although confidence and localization quality may decrease when targets are very small or substantially occluded.

### 2.4. Spatiotemporal Modeling and Risk

To extend beyond detection, the proposed method introduces a spatiotemporal risk modeling module that quantifies avian activity in terms of ecological relevance. Rather than relying solely on instantaneous detections, the model integrates temporal frequency and species-specific characteristics to better reflect real-world behavioral dynamics. For each time step t, a normalized risk score $R_{t}$ is defined as:

(3) $$R_{t}\;=\;\alpha \cdot \frac{N_{t}}{N_{max}}\;+\;\beta \cdot \frac{F_{t}}{F_{max}}\;+\;\gamma \cdot S_{t}$$

where $N_{t}$ represents the number of detected individuals, $F_{t}$ denotes the frequency of species occurrence within a defined temporal window, and $S_{t}$ is a species-specific weighting factor reflecting ecological or operational impact. The normalization terms $N_{max}$ and $F_{max}$ ensure that each component is scaled appropriately, preventing dominance by any single variable and enabling consistent comparison across different observation conditions. *N_max_* represents the maximum observed population count used to normalize the current count $N_{t}$. *N_max_* = 7, corresponding to the highest number of individuals detected at any evaluated time point; therefore, this value was derived from the observations rather than manually assigned. For deployment at other sites or monitoring periods, *N_max_* should be recalculated from the corresponding observation set or defined from an empirically established site-specific upper bound. Similarly, *F_max_* represents the maximum occurrence-frequency value obtained within the selected temporal window ΔT and is used to normalize $F_{t}$. Thus, both normalization parameters are determined from the relevant monitoring data and may vary according to the observation window and site conditions. The coefficients α, β, and γ are tunable parameters that control the relative contribution of each component. The weighting coefficients α = 0.4, β = 0.3, and γ = 0.3 were selected as illustrative values to demonstrate the operation of the Decision-AI framework, with slightly greater emphasis assigned to the primary ecological-risk component. These coefficients were not derived through grid-search optimization or experimental calibration and should therefore not be interpreted as universally optimal values.

This formulation enables the system to capture not only instantaneous detections but also temporal trends and species dominance patterns, which are critical for understanding avian behavior in aquaculture environments. By transforming raw detection outputs into a structured risk indicator, the approach provides a more meaningful representation of avian activity that can support downstream decision-making. The species-specific weighting factor ($S_{t}$) represents the relative ecological and operational impact associated with each detected species. Higher values are assigned to species that are known to exert greater predation pressure on aquaculture stocks, whereas lower values are assigned to species that primarily represent nuisance activity or non-predatory observations. The weighting scheme used in this study was derived from documented avian interactions in aquaculture environments and is intended to provide a practical mechanism for incorporating species relevance into the ecological risk assessment process. The weighting factors presented in Table 1 allow the risk model to account for differences in species-specific ecological importance beyond simple detection frequency. The weighting factors presented here are illustrative and are intended to demonstrate how species-specific ecological relevance can be incorporated into the risk model. They should not be interpreted as empirically validated estimates of predation impact. For example, repeated detections of Great Blue Herons contribute more strongly to the overall risk score than equivalent detections of Canada Geese due to their higher predation potential. Similarly, human observations are assigned a weight of zero because they serve as a non-predatory reference class within the monitoring system. By incorporating ecological relevance into the risk computation, the proposed Decision-AI system can generate more meaningful risk estimates and support adaptive, species-specific, and non-lethal management decisions.

To explicitly capture the temporal dynamics of avian activity, the frequency term $F_{t}$ is computed using a sliding time window. This formulation accounts for repeated detections over a defined interval, allowing the system to distinguish between isolated events and sustained activity patterns:

(4) $$F_{t}\;=\;\frac{1}{\Delta T}\sum_{i\;=\;t\;-\;\Delta T}^{t}\;\;N_{i}$$

where $F_{t}$ represents the normalized frequency of detections at time t, $N_{i}$ denotes the number of detected individuals at each time step i, and ΔT defines the length of the selected observation window. This formulation enables the model to incorporate short-term temporal history, expressed in the temporal unit used for monitoring records, and *t* denotes the discrete observation index.

By integrating this temporal component into the overall risk formulation, the framework is able to differentiate between sporadic detections and consistent behavioral patterns. This distinction is particularly important in aquaculture environments, where repeated visits within a short time frame may indicate elevated interaction levels and increased operational risk.

### 2.5. Example Risk Score Computation

To illustrate the operation of the proposed ecological risk model, consider a hypothetical observation period in which five individuals of a predatory species were detected within the monitoring interval. For demonstration purposes, the example uses the illustrative species-specific weighting factors defined in Table 1. These values are used to demonstrate the computational behavior of the proposed framework and do not represent field-calibrated ecological risk coefficients. Assuming a normalized population contribution of $\frac{N_{t}}{N_{max}}\;=\;0.5$, a temporal activity score of ${F_{t}/F}_{max}\;=\;0.75$, and a species-specific weighting factor of $S_{t}\;=\;1.0$ corresponding to a high-impact predatory species, the ecological risk score can be estimated using the proposed formulation. For this example, the weighting coefficients were set to α = 0.4, β = 0.3, and γ = 0.3.

(5) $$R_{t}=0.4\left(0.5\right)+0.3\left(0.75\right)+0.3\left(1.0\right)\;\;\;\;\;\;\;\;\;R_{t}=0.725$$

Based on the risk categories defined in Table 1, the resulting score corresponds to a high-risk condition, indicating that the observed activity may warrant targeted management attention. This example demonstrates how population abundance, temporal occurrence patterns, and species-specific ecological relevance are jointly incorporated into the proposed Decision-AI system.

### 2.6. Ecological Risk Categorization

To facilitate interpretation of the computed risk scores, the proposed approach classifies avian activity into three ecological risk levels: low, moderate, and high. Low-risk conditions generally correspond to infrequent detections, low population abundance, or species with limited ecological impact. Moderate-risk conditions indicate increased activity levels that may warrant continued observation and monitoring. High-risk conditions represent elevated ecological concern resulting from frequent detections, increased abundance, or the presence of species associated with substantial predation pressure. This categorization provides an intuitive mechanism for translating quantitative risk scores into actionable ecological intelligence and serves as the basis for subsequent Decision-AI and adaptive management processes.

### 2.7. Decision-AI Layer for Non-Lethal

Building upon the computed risk scores, the system incorporates a Decision-AI layer that translates ecological indicators into actionable insights. Rather than applying uniform deterrence strategies, the proposed system enables species-specific and adaptive intervention based on observed activity patterns. To formalize the decision-making process, a threshold-based function is defined to determine whether intervention is required at a given time step:

(6) $$D_{t}\;=\;\{1,\;R_{t}\;\geq \;\tau \;0,\;R_{t}\;<\;\tau \;$$

where $D_{t}$ represents the decision state at time t, with a value of 1 indicating that an intervention is triggered and 0 indicating no action. The parameter τ denotes a predefined risk threshold that controls the sensitivity of the system.

This formulation enables the system to move beyond passive monitoring toward automated, data-driven decision-making, where responses are triggered only when activity exceeds a meaningful level of ecological or operational concern.

To further support non-lethal and species-specific management, the decision process is extended to a multi-level action model:

(7) $$A_{t}\;=\;\{Targeted\;deterrence,\;R_{t}\;\geq \;\tau_{h}\;Monitoring\;only,\;\tau_{l}\;\leq \;R_{t}\;<\;\tau_{h}\;No\;action,\;R_{t}\;<\;\tau_{l}\;$$

where $A_{t}$ represents the selected management action, and $\tau_{h}$ and $\tau_{l}$ define upper and lower decision thresholds, respectively. This hierarchical formulation enables:

- Species-specific intervention timing;
- Adaptive deterrence scheduling;
- Non-lethal response prioritization.

For example, high-risk conditions during specific time windows can trigger targeted and time-sensitive deterrence, while moderate activity levels may require continued monitoring. Low-risk conditions result in no intervention, thereby reducing unnecessary disturbance to non-target species and supporting ecological balance. This component represents a key advancement over conventional monitoring systems by transforming passive observation into active, decision-driven management support. The parameters τ, $\tau_{h}$, and $\tau_{l}$ are tunable decision thresholds rather than fixed biological constraints. In this study, illustrative values were used to demonstrate the framework operation; future deployment should estimate these parameters from site-specific field data and management outcomes. To facilitate practical implementation, risk scores are further categorized into management levels as shown in Table 2. For the present methodological demonstration, the decision thresholds were defined as illustrative values to translate the normalized risk score into low, moderate, and high management categories. These boundaries were not empirically derived from measured predation rates or production-loss data and therefore should not be interpreted as universally validated ecological thresholds.

Based on the assigned risk category, the system recommends one of three adaptive management actions. Low-risk conditions require no intervention, thereby avoiding unnecessary disturbance to wildlife. Moderate-risk conditions trigger continued monitoring to evaluate potential changes in activity patterns. High-risk conditions initiate targeted deterrence measures aimed at reducing predation pressure while minimizing impacts on non-target species. In operational applications, these thresholds should be calibrated using site-specific field observations and management objectives rather than applied as fixed universal values. In this study, targeted deterrence refers to the selective application of a non-lethal response at the relevant time and location when bird activity exceeds the defined high-risk threshold. Depending on farm infrastructure, this recommendation may be implemented manually by farm personnel or, in future deployments, linked to acoustic, visual, water-based, or other site-appropriate deterrent devices. The present study evaluates the detection, risk-assessment, and decision-support components of this process. It does not experimentally validate autonomous deterrent activation or quantify reductions in bird predation following intervention. Therefore, the outputs should be interpreted as management recommendations indicating when and where non-lethal intervention may be warranted.

Operational pseudocode of the Decision-AI Workflow is presented below as an example (Algorithm 1):
**Algorithm 1**. Spatiotemporal Decision-AI workflowInput: Image I Timestamp T Camera/location ID L Species weights Ws Decision thresholds τl and τhOutput: Species detections Population count Ecological risk score R Management recommendationSteps:1. Acquire image I and associated metadata (T, L).2. Apply YOLOv8 detector to I.3. For each accepted detection:   record class label,   confidence score,   and bounding-box coordinates.4. Apply non-maximum suppression.5. Count accepted detections for each target species.6. Retrieve temporal and spatial metadata associated with the image.7. Update species occurrence frequency and recurrence information.8. Assign the corresponding species-specific ecological weight Ws.9. Combine population count, temporal/spatial information,   recurrence, and ecological weighting to compute normalized risk score R.10. If R ≤ τl:  recommendation = “No Action” Else if τl < R ≤ τh:  recommendation = “Monitoring” Else:  recommendation = “Targeted Deterrence”11. Store detection, metadata, risk score, and recommendation.12. Return results.

### 2.8. Feature-Region Visualization and Interpretation

To provide a qualitative interpretation of the visual characteristics associated with the target classes, representative feature-region visualizations were generated from selected images. These visualizations highlight anatomically relevant regions such as the head and neck, body, wings, legs, and overall silhouette that are commonly useful for distinguishing among the monitored classes. The colored regions shown in presented are intended as qualitative anatomical-region annotations rather than continuous class-activation heatmaps. Accordingly, these visualizations should not be interpreted as Grad-CAM, saliency-map, or other quantitative attribution outputs. Their purpose is to illustrate representative morphological regions relevant to species and Human-class differentiation and to support qualitative interpretation of the image characteristics associated with model classification.

## 3. Results

The experimental results demonstrate that the proposed system achieves strong performance not only in species detection but also in enabling spatiotemporal ecological interpretation for decision-oriented analysis.

The classification results (Table 3) show consistently high precision, recall, and F1-scores across all target classes, indicating that the detection model can reliably distinguish between similar avian species under variable field conditions. The balanced performance across species suggests robustness to variations in morphology, posture, and environmental background.

Between widely used pretrained architectures tested, including ResNet50, VGG16, MobileNetV2, and EfficientNet-B0, the proposed YOLOv8-based framework achieved the highest overall accuracy of 97.2%, along with strong precision and recall values (Table 4). This result supports its use as the visual detection component of the broader framework, while the methodological contribution of the study remains focused on the integration of detection outputs with spatiotemporal metadata, ecological risk assessment, and management-oriented decision support. The 5-fold cross-validation analysis showed that the YOLOv8-based detector achieved the highest mean accuracy of 97.20%**,** with corresponding mean precision, recall, and F1-score values of 96.26%, 95.80%, and 96.00%, respectively. EfficientNet-B0 achieved the second-highest mean accuracy (95.66%), followed by MobileNetV2 (94.58%), ResNet50 (93.52%), and VGG16 (91.80%). The variability of model performance across folds was summarized using the standard deviation of each metric. Statistical comparison among the evaluated models was performed using the Friedman test to determine whether the observed differences in performance were statistically significant. Because the evaluation was based on a single grouped partition, the observed differences among models should be interpreted as comparative performance under the present experimental configuration rather than as statistically confirmed differences across repeated dataset partitions. While lightweight models such as MobileNetV2 and EfficientNet-B0 demonstrated competitive performance, they exhibited slightly lower precision–recall balance under field conditions. In contrast, deeper architectures such as VGG16 and ResNet50 showed reduced effectiveness, likely due to limitations in capturing fine-scale spatial features associated with small or partially occluded bird targets.

The training and validation accuracy curves (Figure 4) demonstrate a steady improvement throughout the optimization process, with both curves converging toward approximately 100% accuracy. The close agreement between training and validation performance indicates strong generalization capability and suggests that the YOLOv8-based detection model used within the proposed Decision-AI framework successfully learned discriminative species-specific features without exhibiting significant overfitting. The stable convergence behavior further supports the suitability of the selected network architecture and training configuration for avian species recognition under field conditions.

The training and validation loss curves (Figure 5) exhibit a smooth and consistent decrease throughout the learning process, eventually stabilizing at low loss values. The close correspondence between the two curves indicates stable optimization and further confirms the absence of substantial overfitting. Together with the accuracy results, these findings demonstrate that the proposed method effectively learned representative visual features required for reliable species classification and population estimation under diverse environmental conditions.

**Figure 6:**
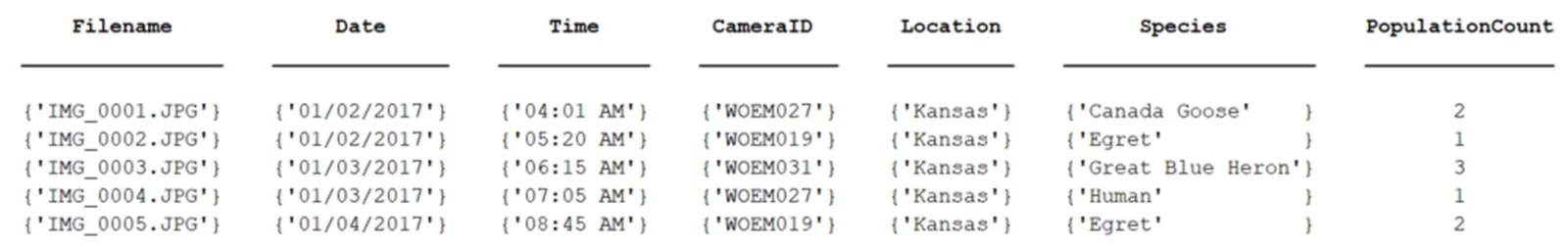
Representative structured ecological database produced by integrating species detection, population estimation, and metadata extraction. Each observation record contains species identity, abundance, timestamp, camera location, and contextual information that supports spatiotemporal profiling, ecological risk assessment, and Decision-AI-based management recommendations.

Output from the model can be formatted into a structured ecological database generated by integrating species detection, population estimation, and metadata extraction within the proposed system (Figure 6). Each observation record contains species identity, population count, timestamp, camera identifier, and location information, enabling detections to be linked to specific spatial and temporal contexts. This demonstrates the success of metadata extraction of the models. By transforming individual image-level predictions into structured ecological records, the system establishes the foundation for spatiotemporal profiling, ecological risk assessment, and adaptive decision-making.

**Figure 7:**
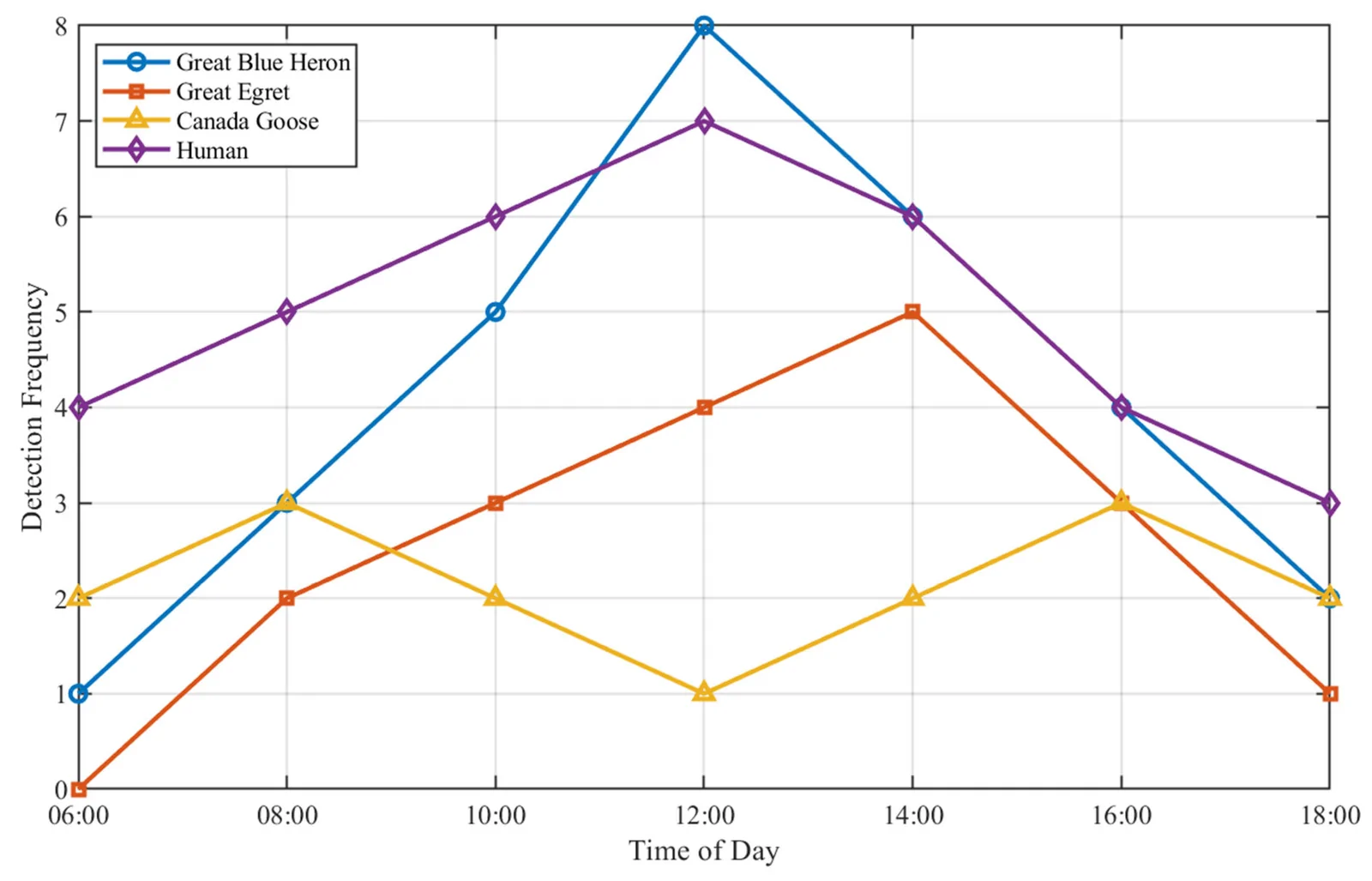
Illustrative species-specific temporal activity profiles generated from aggregated detection records. Detection frequency is summarized across observation periods for Great Blue Heron, Great Egret, Canada Goose, and Human classes.

The proposed spatiotemporal integration system enables temporal activity analysis by aggregating species detections over predefined observation windows. Detection frequency distributions can be used to characterize visitation intensity, identify peak activity periods, and quantify species-specific occurrence trends. As an example, different species exhibit distinct temporal occurrence patterns throughout the monitoring period (Figure 7). Such information enables the identification of peak activity windows, recurring visitation behavior, and periods of elevated ecological interaction. These temporal indicators form the basis of the temporal activity function ($F_{t}$) and contribute directly to the ecological risk assessment and Decision-AI processes described.

To demonstrate the application of the proposed Decision-AI framework, the great blue heron and Canada goose are used as examples, as each represents a different level of predation as determined by species-specific ecological weight in Table 1. In Table 5, N_t_ is the population count detected by the camera at each timepoint (t). The R_t_ score has been calculated for two different time windows where ΔT = 6 or ΔT = 4. These time points can be assigned by researchers and these will generally slide across the dataset to capture new timepoints as data arrives. For demonstration purposes, the time points do not slide, but instead are definite here. The same weighting coefficients are used for α = 0.4, β = 0.3, and γ = 0.3 as in Equation (5). F_t_ is first calculated for each timepoint using Equation (4). F_max_ for the ΔT of interest is then used in Equation (3), along with N_t_ and N_max_ (N_max_ = 7 for both ΔT in this case) for calculating R_t_. Risk category is then assigned as previously described in Table 2.

**Figure 8:**
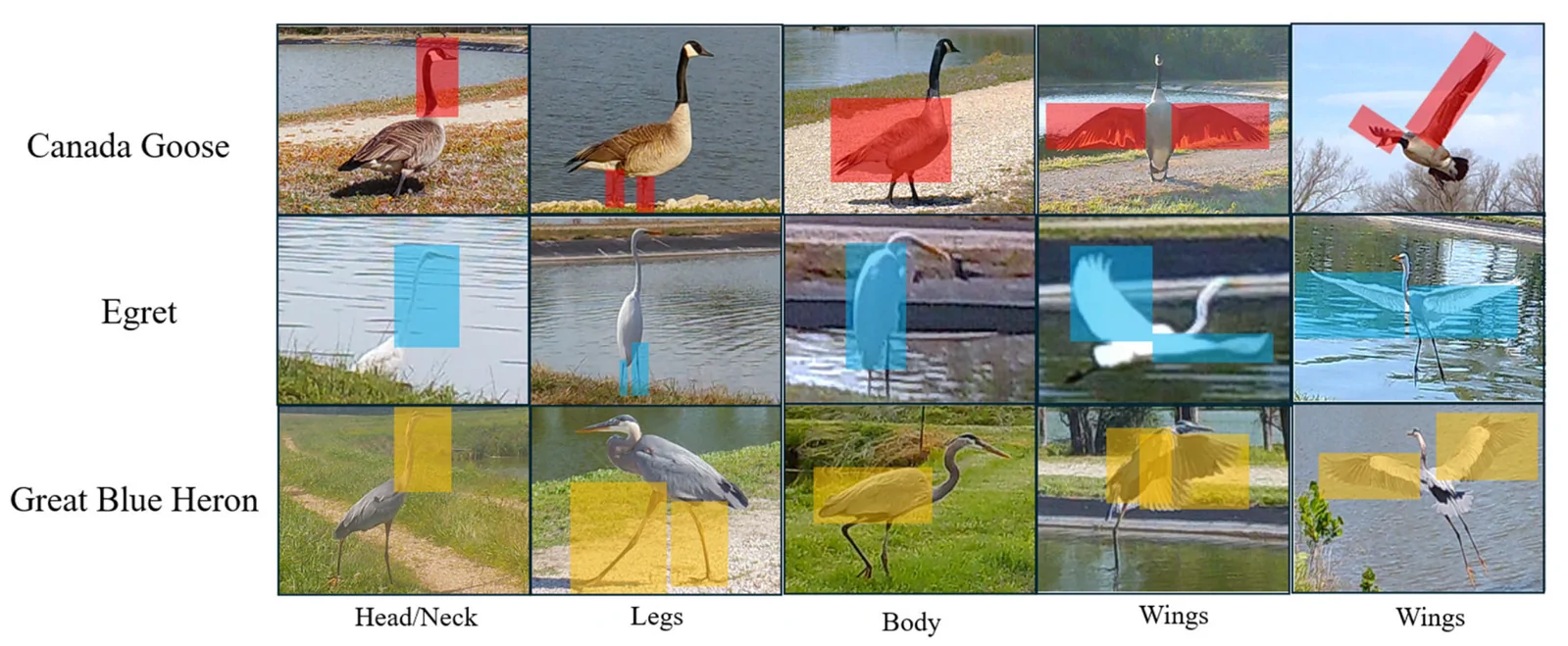
Representative anatomical feature-region visualizations for the avian target classes. Colored regions indicate morphological characteristics commonly associated with class differentiation, including head-neck structure, body contour, leg morphology, and wing regions. The overlays are provided for qualitative interpretation and do not represent continuous class-activation or saliency heatmaps.

**Figure 9:**
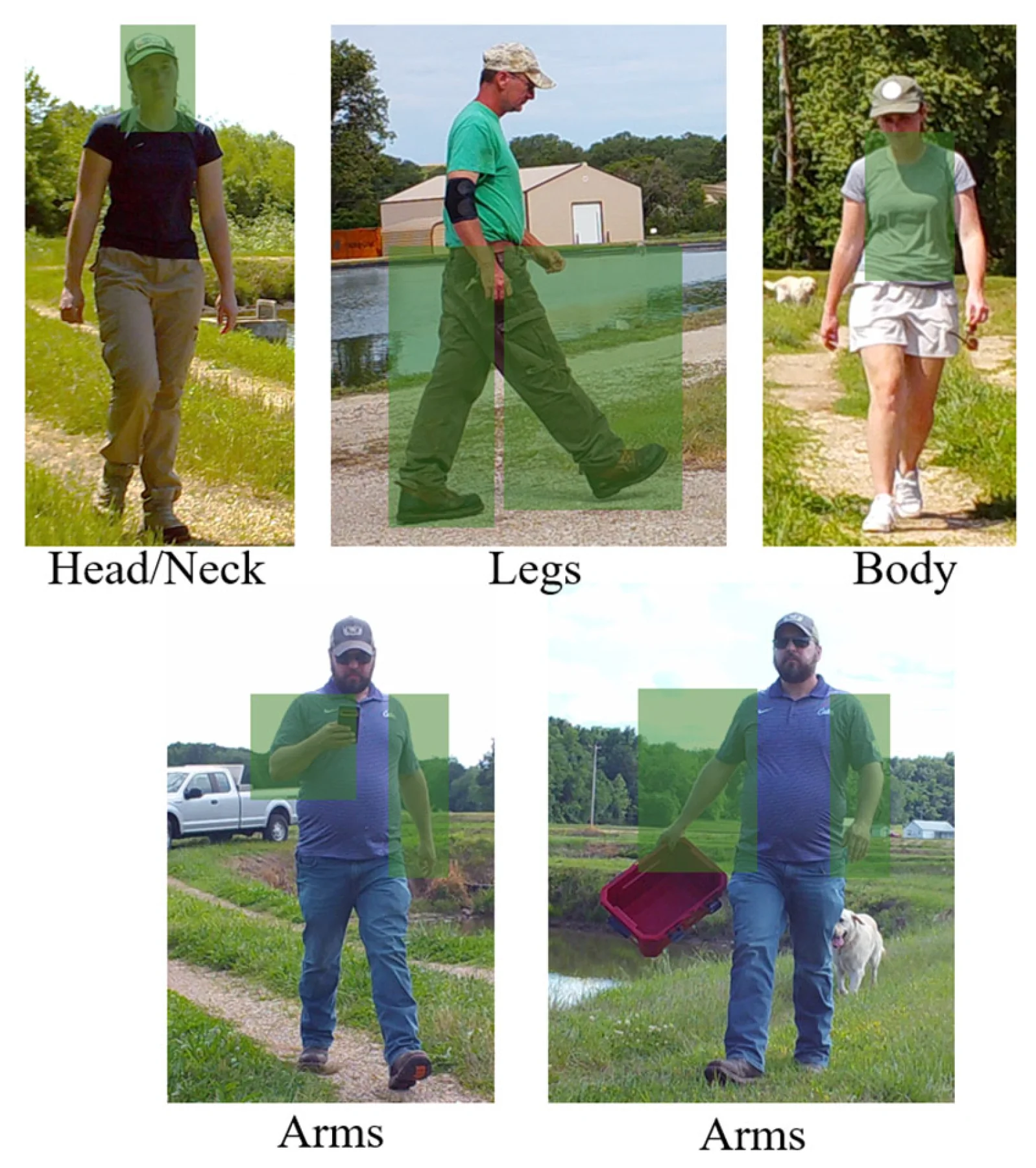
Representative anatomical feature-region visualizations for the Human reference class. Colored regions indicate body areas such as the head-neck region, torso, arms, and legs that illustrate morphological differences from the avian target classes. The overlays are qualitative region annotations rather than continuous model-attribution heatmaps.

Representative feature-region visualizations are presented in Figure 8 and Figure 9 to illustrate anatomical characteristics relevant to differentiation among the target classes. For the avian classes, these include the head-neck region, body contour, wing structure, leg morphology, and overall posture. For the human reference class, relevant visual characteristics include body silhouette, torso structure, and limb geometry. These examples provide a qualitative illustration of morphological differences among the monitored classes; however, they are not intended as quantitative attribution maps of the internal decision process of the YOLOv8-based detector.

Across all evaluated bird species, the model consistently emphasized morphological characteristics commonly used in field identification. For Canada goose observations, attention was concentrated on the head-neck region, body contour, and wing structures. Similar activation patterns were observed for egrets, where elongated neck morphology and wing features received strong attention. In great blue herons, the model focused on distinctive body posture, leg proportions, and neck structures. These results indicate that the proposed system learned biologically meaningful species-specific features rather than relying primarily on environmental background information, thereby improving confidence in the ecological reliability and interpretability of the classification process. In addition, representative explainable AI visualizations for the human reference class, highlighting the anatomical regions that contributed most strongly to classification decisions (Figure 9). The model primarily focused on body posture, limb geometry, torso structure, and overall human silhouette characteristics. These activation patterns indicate that the proposed system learned discriminative human-specific features rather than relying on environmental background information. Such feature extraction improves the interpretability of the model and enhances its ability to distinguish human observations from avian species, thereby reducing potential misclassification in real-world aquaculture environments.

## 4. Discussion

Automated identification of avian species associated with aquaculture environments can support more efficient monitoring and more selective management of wildlife interactions. In the present study, the YOLOv8-based detection model distinguished two predatory avian species, great blue heron and great egret, one primarily non-predatory nuisance avian species, Canada goose, and a human reference class. The model achieved high classification performance, supporting its use as the visual-detection component of the proposed spatiotemporal Decision-AI framework. The contribution of the present study should be interpreted primarily at the framework level rather than as a modification of the YOLOv8 architecture. YOLOv8 functions as the visual detection module, while the proposed framework extends conventional detection outputs by integrating species identity, population abundance, temporal and spatial metadata, occurrence frequency, ecological weighting, and risk-oriented management interpretation. The objective is therefore not to introduce a new detector architecture, but to demonstrate how computer-vision outputs can be transformed into contextual ecological information for aquaculture decision support.

This direction is consistent with recent advances in AI-enabled ecological monitoring. Song et al. [15] evaluated multiple deep-learning object detectors for wild-bird recognition in complex forest scenes and demonstrated that bird detection remains challenging when targets are small, visually concealed, or embedded in complex natural backgrounds. Their results also showed that detector architecture and backbone selection can substantially influence recognition performance. Fergus et al. [16] extended automated camera-trap analysis beyond species recognition by integrating visual AI with contextual ecological information to support richer biodiversity assessments across temporal and spatial dimensions.

Other recent systems have focused on real-time ecological surveillance and resource-efficient deployment. The EcoWatch framework combined YOLOv8-based bird detection and counting with wireless sensor-network communication and environmental context awareness for migratory-bird surveillance [17]. Its emphasis was on real-time monitoring, transmission efficiency, and adaptation to variable environmental conditions. In aquaculture, Bose et al. [11] combined IoT-based environmental monitoring with PIR-based motion detection and automated water spraying to reduce bird attacks on fish ponds. That system demonstrates the feasibility of linking sensing directly to non-lethal deterrence, but the deterrence mechanism was triggered by motion detection rather than species-specific visual recognition or ecological risk differentiation. The present framework differs from these approaches by introducing an intermediate ecological decision layer between visual detection and management response (Table 6). Species identity and population estimates are combined with temporal recurrence, spatial information, and species-specific ecological relevance before a management recommendation is generated. Thus, the proposed Decision-AI framework is intended to move beyond isolated detection events toward contextual interpretation of when, where, and how strongly avian activity may warrant management attention. Rather than using detection solely to identify wildlife, enrich ecological reports, or directly trigger a generic deterrent response, the proposed approach introduces an intermediate ecological interpretation layer that distinguishes isolated observations from recurring or higher-risk activity before a management recommendation is generated. The methodological contribution therefore lies primarily in the integration of visual monitoring with spatiotemporal ecological risk interpretation and decision support.

The integration of metadata is central to this transition. A single visual detection provides limited ecological context, whereas linking observations with time, location, species identity, population count, and recurrence allows the framework to distinguish isolated events from repeated or concentrated activity. This supports the transition from image-level recognition toward spatiotemporal ecological assessment and management-oriented interpretation. The species-specific weighting factors used in the present framework should be interpreted as illustrative parameters rather than empirically validated ecological coefficients. Their purpose is to demonstrate how differences in species-specific ecological relevance can be incorporated into the risk calculation. A formal sensitivity analysis of the weighting coefficients was not performed in the present study. Consequently, the extent to which alternative values of α, β, and γ may influence the normalized risk score and resulting management category remains to be quantified. Future work should systematically vary these coefficients while maintaining α + β + γ = 1 and evaluate their effects across representative field scenarios. Field-based calibration using observed predation events, abundance, recurrence, and management outcomes will be necessary to establish robust site-specific weighting values using field-based evidence such as species-specific predation frequency, duration of aquaculture interactions, observed fish losses, and other site-specific operational impacts. Such calibration would strengthen the biological interpretation of the resulting risk scores and improve adaptability across different aquaculture systems.

Similarly, the decision thresholds used to classify risk as low, moderate, or high are illustrative rather than field-validated management cutoffs. The present 0.30 and 0.70 boundaries were selected to demonstrate how a continuous normalized risk score can be translated into discrete management-oriented categories. Future validation should calibrate these thresholds against observed species activity, population abundance, confirmed predation events, fish losses, and management outcomes. Threshold values may ultimately require adjustment according to local species composition, facility configuration, production scale, and operational risk tolerance. Within the proposed Decision-AI framework, low-risk conditions correspond to No Action, moderate-risk conditions to Monitoring, and high-risk conditions to Targeted Deterrence. These outputs should be interpreted as decision-support recommendations rather than validated intervention outcomes. Targeted Deterrence refers to the selective application of a site-appropriate non-lethal response at the relevant time and location when activity exceeds the defined high-risk threshold. Such a response could be implemented manually by farm personnel or, in future deployments, linked to acoustic, visual, water-based, or other non-lethal deterrent devices. The present study did not experimentally determine whether implementation of a Targeted Deterrence recommendation reduces bird visitation, predation pressure, or fish loss. Controlled field trials will therefore be necessary to determine whether risk-informed intervention improves deterrence effectiveness.

Environmental variability is another important consideration. Outdoor aquaculture imagery may be influenced by illumination changes, reflective water surfaces, vegetation, partial occlusion, target scale, and background complexity. These challenges are consistent with those reported in recent wild-bird detection studies, where small targets and complex ecological backgrounds can reduce recognition performance. Although the close training and validation accuracies observed in the present study indicate stable convergence under the current dataset configuration, they should not be interpreted as evidence of complete robustness to all outdoor conditions. Future evaluation should include condition-specific and cross-site testing to quantify performance under different environmental scenarios. The grouped 70:30 partitioning strategy was used to reduce the possibility that closely related observations from the same camera location or capture session were distributed across both training and testing subsets. This approach reduces the risk of leakage from temporally or spatially correlated observations and provides a more realistic estimate of performance on independent field observations. Nevertheless, a single train-test partition does not fully quantify variability across alternative partitions. Future work should therefore include grouped k-fold cross-validation, repeated partitioning, or cross-site validation to provide a more comprehensive assessment of model generalization.

The existing benchmark models, including ResNet50, VGG16, MobileNetV2, and EfficientNet-B0, provide useful convolutional baselines but do not fully represent the most recent object-detection landscape. More recent architectures such as YOLOv10, YOLO11, RT-DETR, and DINO provide relevant detector and Transformer-based alternatives for future comparison. These architectures were not retrained on the present dataset and should therefore be regarded as literature-based contextual benchmarks rather than direct experimental baselines. Recent detector families provide useful points of comparison with the YOLOv8-based module used in this study. YOLOv10 introduced an end-to-end, NMS-free detection strategy through consistent dual assignments, with the aim of improving the efficiency–accuracy trade-off for real-time detection [19]. YOLO11 further refined the Ultralytics detection family through improved backbone and neck components, attention-based feature processing, and greater parameter efficiency relative to YOLOv8 [20]. In contrast, RT-DETR represents a Transformer-based end-to-end detector designed for real-time applications, using an efficient hybrid encoder and query-selection strategy while avoiding conventional NMS post-processing [21]. DINO, another DETR-family architecture, improves Transformer-based detection through denoising training, improved query initialization, and iterative box refinement. These architectures illustrate recent alternatives to conventional CNN-based and YOLO-style detectors and provide relevant benchmarks for future aquaculture-specific evaluation. Because they were not retrained on the present dataset, however, their reported performance cannot be directly compared numerically with the YOLOv8 results obtained here. A controlled comparison using the same dataset, grouped partitioning strategy, and evaluation metrics is therefore recommended for future work.

In addition, computational efficiency must be evaluated for practical edge deployment by characterizing parameter count, floating-point operations (FLOPs), frames per second (FPS), memory requirements, and hardware configuration together with detection performance to better calculate computation–accuracy trade-offs. For example, higher-capacity detectors may provide improved feature representation and localization performance, but they typically require greater computational resources, memory, and inference time. In contrast, more computationally efficient models may be better suited to embedded or edge platforms, particularly when real-time response is required. Accordingly, the suitability of the YOLOv8-based detector for field deployment should be interpreted in relation to its parameter count, FLOPs, inference speed, and detection accuracy rather than accuracy alone. This computation–accuracy trade-off is especially important for aquaculture monitoring systems intended for continuous operation on resource-constrained edge devices. Future deployment studies should therefore evaluate model optimization strategies such as pruning, quantization, and hardware-specific acceleration while monitoring any associated loss in detection performance.

Overall, the findings demonstrate the potential value of integrating computer vision with spatiotemporal ecological context and management-oriented reasoning. Rather than treating detections as isolated outputs, the proposed Decision-AI framework retains information on species identity, abundance, location, time, recurrence, and ecological relevance to support risk-oriented interpretation. The present work should nevertheless be regarded as an initial decision-support framework rather than a fully validated autonomous deterrence system. This approach aligns with the increasing need for sustainable and ecologically responsible aquaculture practices. By minimizing unnecessary interventions and focusing on high-risk conditions, the system promotes both operational efficiency and environmental stewardship.

## 5. Conclusions

This study presented a spatiotemporal Decision-AI system for species-specific and non-lethal management of avian predators in aquaculture environments. By integrating image preprocessing, structured metadata extraction, and deep learning–based detection using YOLOv8, the proposed system enables reliable identification of bird species and estimation of population counts under real-world field conditions. Beyond conventional monitoring, the method extends detection capabilities through the integration of spatial and temporal context, allowing the characterization of avian activity patterns across locations and time. This enables a transition from isolated visual observations to structured ecological intelligence, where species presence, frequency, and distribution can be systematically analyzed.

A key contribution of this work lies in bridging the gap between visual detection and decision-oriented analysis. By incorporating spatiotemporal information, the proposed approach supports the identification of activity trends and potential risk conditions, providing a foundation for species-specific and non-lethal management strategies. Rather than applying generalized deterrence, the system enables more targeted and adaptive responses that minimize unnecessary disturbance to non-target wildlife while maintaining aquaculture productivity. The experimental results demonstrate consistent detection performance across multiple locations, confirming the robustness of the approach under varying environmental conditions. More importantly, the integration of detection outputs with contextual metadata highlights the potential of AI systems to move beyond passive monitoring toward data-informed ecological decision support.

Several limitations should be considered when interpreting the present findings. First, although a grouped 70:30 training–testing split was used to reduce potential data leakage, evaluation based on a single partition does not fully quantify variability across alternative dataset partitions or independent sites. Second, environmental factors such as illumination, reflective water surfaces, occlusion, target scale, and background complexity were naturally represented in the dataset but were not systematically controlled or labeled as independent experimental variables. Third, the species-specific ecological weighting factors and the low, moderate, and high risk thresholds were used as illustrative parameters to demonstrate framework operation and were not empirically calibrated against measured predation rates, fish losses, or management outcomes. In addition, the feature-region visualizations were qualitative anatomical annotations rather than formal explainability outputs such as Grad-CAM or saliency maps. The current framework was also evaluated using a limited set of target classes and study locations, which may restrict broader generalization. In addition to precision, recall, and AP at an IoU threshold of 0.50, stricter localization metrics such as AP@0.75 and mAP@0.5:0.95 would provide a more comprehensive assessment of bounding-box localization quality. These metrics were not included in the original evaluation and should be incorporated in future validation using the same grouped dataset protocol.

Finally, the Decision-AI outputs should be interpreted as management-oriented recommendations rather than validated deterrent outcomes. The present study did not experimentally evaluate whether Targeted Deterrence reduces bird visitation, predation pressure, or production loss. Future work should therefore include grouped k-fold or repeated validation, independent cross-site testing, condition-specific environmental analysis, empirical calibration of ecological weights and risk thresholds, formal explainability methods, expansion to additional species and locations, and controlled field trials linking Decision-AI recommendations to actual non-lethal deterrent responses.

Future work will focus on expanding species coverage, incorporating additional environmental variables, and further developing the decision-making component through multi-modal sensing integration. In addition, developing the target scale, particularly when birds appear distant or occupy only a small portion of the image will help refine implementation of deterrent systems. Precision separately for small (APs), medium (APm), and large (APl) objects using standardized area-based categories would provide a clearer assessment of the detector’s sensitivity to objective size under field conditions. These directions aim to enhance the system’s capability to support adaptive, real-time, and ecologically sustainable aquaculture management while reducing unintended environmental impacts.

## Figures and Tables

**Table 1:** Illustrative species-specific ecological weighting factors used to demonstrate the proposed risk-assessment framework. The values are relative to the target classes included in this study and were not empirically calibrated against measured predation or production-loss data.

Species	Weight (*S_t_*)	Ecological Rationale
Great Blue Heron	1.0	Major fish predator frequently associated with direct predation losses
Great Egret	0.8	Predatory species with moderate to high interaction potential
Canada Goose	0.6	Primarily nuisance activity with limited direct predation impact
Human	0.0	Non-predatory reference class used for contextual monitoring

**Table 2:** Illustrative decision thresholds used to translate normalized ecological risk scores into management-oriented categories. The threshold boundaries are provided for methodological demonstration and were not empirically calibrated against field-measured predation or production-loss data.

Risk Score ($R_{t}$)	Risk Category	Recommended Action
0.00–0.30	Low	No Action
0.31–0.70	Moderate	Monitoring
0.71–1.00	High	Targeted Deterrence

**Table 3:** Species-wise performance metrics of the YOLOv8-based detection model used within the proposed spatiotemporal Decision-AI framework. Precision, recall, and F1-score indicate classification performance across the avian and human reference class.

Species	Precision (%)	Recall (%)	F1-Score (%)
Egret	95.6	96.0	95.8
Great Blue Heron	96.3	95.7	96.0
Canada Goose	95.1	96.5	95.8
Human	96.2	97.5	96.8

**Table 4:** Model Comparison Summary.

Model	Mean Accuracy (%)	Precision (%)	Recall (%)	F1-Score (%)
Proposed Model	97.2	96.4	95.8	96.00
ResNet50	93.5	91.7	92.1	91.90
VGG16	91.8	90.2	89.4	89.80
MobileNetV2	94.6	93.3	92.7	92.96
EfficientNet-B0	95.7	94.8	94.2	94.46

**Table 5:** Illustrative risk-assessment example for Great Blue Heron and Canada Goose using the demonstration weighting factors defined in Table 1. The calculated values illustrate the operation of the Decision-AI risk model and are not intended as empirically validated predation-risk estimates.

		Great Blue Heron, S_t_ = 1		Canada Goose, S_t_ = 0.6	
t	N_t_	R_t_ and Risk Category, ΔT = 6	R_t_ and Risk Category, ΔT = 4	R_t_ and Risk Category, ΔT = 6	R_t_ and Risk Category, ΔT = 4
1	1	0.37/Moderate	0.38/Moderate	0.25/Low	0.26/Low
2	5	0.66/Moderate	0.71/High	0.54/Moderate	0.59/Moderate
3	7	0.86/High	0.90/High	0.74/High	0.84/High
4	2	0.59/Moderate	0.71/High	0.47/Moderate	0.59/Moderate
5	6	0.89/High	NA	0.78/High	NA
6	4	0.83/High	NA	0.71/High	NA

**Table 6:** Comparison of the proposed framework with representative AI-enabled wildlife aquaculture monitoring approaches.

Study	Application	Main Approach	Context/Metadata	Management Output	Key Distinction from Present Study
Whytock et al. (2023) [18]	Wildlife camera traps	AI classification + satellite alerts	Image label and metadata	Near-real-time alert	Focuses on rapid transmission/notification rather than ecological risk scoring
Fergus et al. (2024) [16]	Biodiversity monitoring	YOLOv10-X + vision–language model	Time, vegetation, distribution and other ecological context	Context-rich ecological reporting	Strong contextual analysis, but not aquaculture-specific risk-to-deterrence decision logic
Bose et al. (2024) [11]	Fish farming	IoT sensors + PIR motion detection + water spray	Water-quality and location information	Direct bird deterrence	Deterrence triggered by motion; no species-specific CV or ecological risk layer
EcoWatch (2025) [17]	Migratory-bird surveillance	YOLOv8 + counting + LoRaWAN	Environmental/network context	Bird surveillance	Emphasizes detection, counting, and resource-efficient transmission
Present study	Aquaculture avian monitoring	YOLOv8 detection + metadata + spatiotemporal risk model	Species, abundance, time, location, recurrence	No Action/Monitoring/Targeted Deterrence	Integrates visual detection with ecological risk interpretation and management-oriented decision support

## Data Availability

All images used in this study were collected by the authors through dedicated field deployments and experimental testbeds; no publicly available datasets were utilized. The datasets generated and analyzed during this study are available through the USDA Agricultural Research Service (USDA-ARS) Ag Data Commons repository: https://doi.org/10.15482/USDA.ADC/29922011. The repository includes the curated image dataset, associated metadata, and supporting materials necessary to facilitate transparency, reproducibility, and further research. Additional archival resources are preserved on Figshare and are accessible through the following link: https://figshare.com/s/79d8a3e12bc94f4598a3 (accessed on 18 January 2025). These resources provide long-term public access to the data and supporting documentation used in this work, enabling independent validation and future comparative studies.
